# Are older adults living with HIV more susceptible to omicron infection compared to their HIV-negative peers in China: a cross-sectional study

**DOI:** 10.3389/fpubh.2025.1685868

**Published:** 2025-11-05

**Authors:** Jianhui Yang, Esben Strodl, Hong Xu, Haibo Jiang, Kun Chu, Shiwen Tan, Zehao Ye, Hongbo Shi, Weiqing Chen, Feng Tong

**Affiliations:** ^1^Ningbo Municipal Centre for Disease Control and Prevention, Ningbo, China; ^2^School of Psychology and Counselling, Queensland University of Technology, Brisbane, QLD, Australia; ^3^Department of Epidemiology, School of Public Health, Sun Yat-sen University, Guangzhou, China; ^4^Department of Information Management, Xinhua College, Sun Yat-sen University, Guangzhou, China

**Keywords:** HIV, older adults, SARS-CoV-2, omicron, viral shedding, diabetes

## Abstract

**Background:**

Older individuals are particularly susceptible to Omicron infection and long viral shedding durations. However, the associations between human immunodeficiency virus (HIV) status in older people, Severe Acute Respiratory Syndrome Coronavirus 2 (SARS-CoV-2) infection, and viral shedding duration have not been determined. Therefore, this study aims to evaluate the impact of HIV infection status on Omicron infection rates and viral shedding duration in older people.

**Methods:**

This was a cross-sectional study of older adults Chinese participants (aged ≥60 years) who were either people living with HIV (PLWH) or not infected with HIV. A total of 606 participants completed the questionnaire: 226 participants were diagnosed with HIV, and 380 participants reported being HIV-negative. Propensity score matching (PSM) was performed to balance the baseline parameters of the two groups and to exclude the effect of confounding variables, resulting in a final sample of 198 PLWH and 198 HIV-negative participants for data analysis. Risk or protective factors for Omicron infection and a long viral shedding duration, including demographics, HIV-related factors, and comorbidities, were investigated using multivariable logistic regression.

**Results:**

The risk of SARS-CoV-2 infection in the PLWH group was lower than that in the HIV-negative group (odds ratio [OR] = 0.455 [0.285–0.724]). However, PLWH experienced a longer viral shedding duration than the comparison group after Omicron infection (OR = 2.303 [1.221, 4.346]). In addition, older adults with diabetes had higher odds of experiencing a longer viral shedding duration than those without diabetes (OR = 2.742 [1.262, 5.957]). PLWH who had diabetes were at a higher risk of longer viral shedding duration compared with the HIV-negative group (OR = 2.232 [1.132, 4.400]).

**Conclusion:**

Our study demonstrated that older PLWH have decreased odds of being infected with Omicron and experience longer viral shedding duration in comparison to matched HIV-negative controls. Furthermore, diabetes may prolong SARS-CoV-2 viral shedding duration in older people. Targeted monitoring and intervention should be implemented among vulnerable groups, such as older adults individuals infected with HIV or those with underlying diabetes, to prevent and respond to potential outbreaks of SARS-CoV-2 in the future.

## Background

Coronavirus Disease-19 (COVID-19), caused by Severe Acute Respiratory Syndrome Coronavirus 2 (SARS-CoV-2) (1), was first reported in Wuhan, Hubei Province, China, in December 2019 (2). Since the outbreak of this pandemic, COVID-19 has spread rapidly, covering most parts of the world, and has severely affected people’s daily lives and work because of its highly contagious nature and widespread reach (3–5). COVID-19 has been classified as a Public Health Emergency of International Concern (PHEIC) by the World Health Organization (WHO) due to its highly contagious and lethal nature (6). As of January 2024, SARS-CoV-2 has infected over 774 million people and caused more than 7 million deaths worldwide (7). Previous studies have revealed that certain groups, including older adults, males, individuals living with HIV, and those with comorbid conditions such as diabetes, obesity, cardiovascular disease, heart failure, and chronic kidney disease, were at an elevated risk of acquiring SARS-CoV-2 and experiencing fatal outcomes from COVID-19 (8–16). Notably, the older population has borne the brunt of SARS-CoV-2-related infections, hospitalizations, admissions to intensive care units (ICUs), and deaths (17–20). This is primarily attributed to the presence of comorbidities and immunosenescence prevalent among older individuals (21, 22).

During the early stages of the epidemic, preliminary studies appeared to indicate that people living with HIV (PLWH) may be at a higher risk of SARS-CoV-2 infection. However, as the pandemic spread and more extensive research became available, it became increasingly apparent that there was no significant association between the status of human immunodeficiency virus (HIV) and SARS-CoV-2 infection (23–25). It is worth noting that these studies were conducted in younger populations. Given the complex interplay between HIV status, immunological responses, advancing age, and comorbid conditions, there remains considerable uncertainty regarding the comparative susceptibility of older individuals with HIV to the Omicron variant in comparison to their HIV-negative peers (17, 26). Furthermore, there is a limited understanding of whether the time of SARS-CoV-2 viral shedding differs according to HIV status in an older population.

Thus, this study aims to assess the difference in the prevalence rate of Omicron infection and long viral shedding duration between older PLWH and older individuals without HIV. Furthermore, we explored whether the presence of diabetes moderates these associations. Addressing these knowledge gaps is crucial for effective pandemic management and optimization of healthcare strategies for this potentially vulnerable segment of the older population living with HIV.

## Methods

### Study population

A comprehensive cross-sectional survey was conducted in the city of Ningbo, Zhejiang Province, from 15 January to 4 February 2023. This survey employed a simple random sampling approach, specifically targeting two distinct groups: (1) people living with HIV/AIDS aged 60 years or older residing in Ningbo, who were sampled from the HIV/AIDS Prevention and Control Information System of the Chinese Disease Prevention and Control Information System; and (2) HIV-negative individuals aged 60 years or older, drawn from the general population residing in Ningbo. The inclusion criteria involved participants aged ≥ 60 years who had a clear comprehension of the survey’s purpose and significance, demonstrated through the signing of an informed consent form. The exclusion criteria included participants with severe physical illness/disabilities or mental disorders, or those who did not sign an informed consent form. After excluding data from these participants, 606 participants completed the questionnaire. Of this sample, 226 participants were diagnosed with HIV, while 380 participants reported being HIV-negative.

We employed the propensity score matching (PSM) method to select cases and controls from participants who completed the questionnaire (27, 28). To reduce biases and confounding variables of HIV, covariates with *p* < 0.05 between the HIV and HIV-negative groups in the comparative analysis were considered confounding factors. Propensity score matching is a quasi-experimental method that allowed us to construct an artificial control group by matching each HIV-infected patient with a non-HIV-infected individual with similar characteristics. During the PSM process, logistic regression was used to compute a propensity score for each participant, considering variables such as sex, education level (classified as junior high school or lower, high school, and college or above), marital status (single or married), race (Han or Minority), and registered residence (Ningbo or other regions) (29). Subsequently, each HIV-infected patient in the sample was matched with a control subject without HIV infection by employing the nearest neighbor random matching algorithm with a caliper adjustment set at a predetermined width of ±0.05 (30, 31). To evaluate the balance of covariate distribution, the standardized mean difference (SMD) was calculated for each matched variable between PLWH and non-PLWH (31). This approach served as a balanced diagnostic tool for both the precisely matched data and the PSM-adjusted dataset. A smaller SMD indicates a more evenly distributed variable. Typically, an SMD greater than 0.1 is regarded as a sign of imbalance. Consequently, we excluded 28 PLWH and 182 non-PLWH from the analysis, resulting in a final sample of 198 PLWH and 198 HIV-negative individuals, to assess the relationship between HIV status and SARS-CoV-2 infection among older adults in China.

### Data collection and diagnosis

Well-trained health-care assistants helped participants complete a self-administered structured questionnaire (refer to the Supplementary materials for more detailed information regarding the questionnaire), which included (1) sociodemographic characteristics of the individual (e.g., age, sex, marital status, and education level); (2) the presence or absence of hypertension, diabetes, cardiovascular disease, and other morbidities (chronic obstructive pulmonary disease, chronic kidney disease, oncology/cancer, and others); and (3) the participants’ COVID-19 vaccination status (unvaccinated, or one/two doses, or three doses, or four doses (32). The participants were also asked about SARS-CoV-2 infection status between 7 December 2022 and 15 January 2023 (positive SARS-CoV-2 nucleic acid testing, or a positive SARS-CoV-2 rapid antigen test, or uninfected), and investigators checked participants’ health codes, including the results of SARS-CoV-2 nucleic acid testing and SARS-CoV-2 rapid antigen test. According to nationwide surveillance data obtained from the Chinese Center for Disease Control and Prevention (China CDC), genomic sequencing conducted between 26 September 2022, and 23 January 2023, revealed that all SARS-CoV-2 infections during this period were attributed to Omicron variants, specifically the BA.5.2.48 (53.9%), BF.7.14 (25.2%), and BA.5.2.49 (13.4%) sub-lineages (33). Given this consistent epidemiological pattern and based on this question, positive SARS-CoV-2 nucleic acid testing or positive SARS-CoV-2 rapid antigen test was used as the standard diagnostic criterion for Omicron infection in this study.

For the participants who had contracted the Omicron variant, four questions were asked about their health behaviors following infection with SARS-CoV-2: 1. Have you ever sought medical attention since you were infected with SARS-CoV-2? (no, yes); 2. Have you been hospitalized since you were infected with SARS-CoV-2? (no, yes); 3. Have you taken any anti-COVID medications since you were infected with SARS-CoV-2? (no, yes); 4. How many weeks did it take for the COVID-19 antigen or nucleic acid test to become negative, indicating the duration of viral shedding? (less than 2 weeks, more than 2 weeks). For the PLWH participants, data exported from the HIV/AIDS Prevention and Control Information System of the Chinese Disease Prevention and Control Information System included antiretroviral therapy (ART) status (on ART or none), recent CD4 + T lymphocyte count (CD4 count; 0–199 cells/μL, 200–349 cells/μL, 350–499 cells/μL, ≥500 cells/μL, or unknown), and HIV viral load (HIV-VL, undetectable (< 20 IU/mL), detectable (≥ 20 IU/mL), or unknown). Studies involving human participants were reviewed and approved by the Human Research Ethics Committee of the Ningbo CDC in Ningbo, China (no. 201913). Written informed consent to participate in this study was obtained from the participants.

### Confounding variables

Based on previously published literature, the following confounding variables were chosen: age, sex, hypertension, diabetes, cardiovascular disease, and other morbidity (chronic obstructive pulmonary disease, chronic kidney disease, oncology/cancer, and others) (29, 34).

### Statistical analysis

Means and standard deviations (SD) were calculated to describe continuous variables, and frequencies with percentages were calculated to describe dichotomous or categorical variables. Matched pairs were compared using the χ^2^ test for categorical variables and the Mann–Whitney U test or Student’s *t*-test for continuous variables.

To assess multicollinearity, we calculated the Variance Inflation Factors (VIFs), using VIF < 10 as the threshold for an acceptable level of collinearity. To measure the model fit, we calculated R^2^ and AIC/BIC and performed the Hosmer–Lemeshow test. See the results in Supplementary Table 6.

First, multivariate logistic regression analyses with matched pairs were performed to evaluate the association between HIV-infected status and the presence of Omicron infection and the long viral shedding duration among the older adults in China after adjusting for the aforementioned covariates. Second, we employed multivariate logistic regression models to evaluate the association of HIV status with omicron infection or long viral shedding duration after adjusting for the aforementioned covariates. Sensitivity analysis was conducted using ≥3 weeks as the cutoff value of long viral shedding.

Estimated odds ratios (ORs) and their 95% confidence intervals (CIs) were used to quantify the difference in the odds of Omicron infections between PLWH and HIV-negative people. Analyses were performed using R version 4.3.2 (The R Project for Statistical Computing, Vienna, Austria; cran.r-project.org). Statistical significance was defined as a two-sided *p*-value of <0.05.

## Results

### Description of the participants

A summary description of the demographic characteristics of the cases and controls before and after propensity score matching (PSM) is shown in Table 1. Before PSM, significant differences in sociodemographic characteristics between cases and controls were found in sex, marital status, and registered residence. There were no significant differences in education level and race between the cases and controls. After PSM, there were no significant differences in any of the sociodemographic characteristics between the selected cases and controls (Table 1).

**Table 1:** Baseline characteristics of the participating people before and after PSM.

Characteristics	Before PSM (*N* = 606)				After PSM (*N* = 396)			
	HIV-infected status		*P*	SMD	HIV-infected status		*P*	SMD
	PLWH (*N* = 226)	HIV-negative people (*N* = 380)			PLWH (*N* = 198)	HIV-negative people (*N* = 198)		
Age [mean (SD)]	66.86 (5.62)	66.90 (6.13)	0.934	0.007	67.34 (5.55)	66.71 (5.00)	0.232	0.120
Sex (%)			<0.001	0.323			1	0.012
Male	172 (76.1)	233 (61.3)			153 (77.3)	152 (76.8)		
Female	54 (23.9)	147 (38.7)			45 (22.7)	46 (23.2)		
Education level (%)			0.1	0.185			0.893	0.048
Junior high school or lower	187 (82.7)	288 (75.8)			167 (84.3)	166 (83.8)		
High school	29 (12.8)	62 (16.3)			22 (11.1)	21 (10.6)		
College or higher	10 (4.4)	30 (7.9)			9 (4.5)	11 (5.6)		
Marital status (%)			<0.001	0.025			1	<0.001
Single	3 (1.3)	4 (1.1)			3 (1.5)	3 (1.5)		
Married	223 (98.7)	376 (98.9)			195 (98.5)	195 (98.5)		
Race (%)			0.269	0.134			-	<0.001
Han	224 (99.1)	380 (100.0)			198 (100.0)	198 (100.0)		
Minority	2 (0.9)	0 (0.0)						
Registered residence (%)			0.018	0.204			1	0.017
Ningbo	200 (88.5)	358 (94.2)			180 (90.9)	179 (90.4)		
Others	26 (11.5)	22 (5.8)			18 (9.1)	19 (9.6)		

Detailed information on the comorbid medical conditions, vaccination status, and SARS-CoV-2 infection status of the study participants is presented in Table 2. The prevalence of hypertension was higher in PLWH than in HIV-negative individuals. Compared with people who did not contract HIV, PLWH were less likely to receive a COVID-19 vaccination. However, the rate of SARS-CoV-2 infection in the former was significantly higher (76.3% vs. 56.6%, *p* < 0.001) (Table 2).

**Table 2:** The comorbidity and omicron infection status of analytical participants.

Characteristics	HIV-infected status		*P*	SMD
	PLWH (*N* = 198)	HIV-negative people (*N* = 198)		
Hypertension (%)			0.001	0.349
No	149 (75.3)	117 (59.1)		
Yes	49 (24.7)	81 (40.9)		
Diabetes (%)			0.158	0.156
No	174 (87.9)	163 (82.3)		
Yes	24 (12.1)	35 (17.7)		
Cardiovascular disease (%)			0.679	0.062
No	187 (94.4)	184 (92.9)		
Yes	11 (5.6)	14 (7.1)		
Other morbidity (%)			1.000	0.015
No	173 (87.4)	172 (86.9)		
Yes	25 (12.6)	26 (13.1)		
COVID-19 vaccination status (%)			<0.001	0.652
No	53 (26.8)	11 (5.6)		
One dose/two doses	25 (12.6)	32 (16.2)		
Three doses	111 (56.1)	129 (65.2)		
Four doses	9 (4.5)	26 (13.1)		
SARS-CoV-2 infection status (%)			<0.001	0.426
No	86 (43.4)	47 (23.7)		
Yes	112 (56.6)	151 (76.3)		

Furthermore, among those infected with the Omicron variant, more HIV-negative people sought medical attention and took anti-COVID medications compared with PLWH. However, older PLWH had a higher rate of hospitalization and a longer time for the COVID-19 antigen or nucleic acid to turn negative than older HIV-negative people (Table 3). There were significant differences in COVID-19 symptoms, such as runny nose, diarrhea, headaches, and pneumonia, between the two subgroups.

**Table 3:** Behaviors and symptoms of the participants after omicron infection.

Behaviors and symptoms	HIV-infected status		*P*	SMD
	PLWH (*N* = 112)	HIV-negative people (*N* = 151)		
Sought medical attention after SARS-CoV-2 infection (%)			0.103	0.226
No	94 (83.9)	113 (74.8)		
Yes	18 (16.1)	38 (25.2)		
Hospitalization after SARS-CoV-2 infection (%)			0.009	0.35
No	102 (91.1)	149 (98.7)		
Yes	10 (8.9)	2 (1.3)		
Took anti-COVID medications after SARS-CoV-2 infection (%)			<0.001	0.542
No	38 (33.9)	18 (11.9)		
Yes	74 (66.1)	133 (88.1)		
Viral shedding duration (%)			0.002	0.412
< 2 weeks	64 (57.1)	115 (76.2)		
≥ 2 weeks	48 (42.9)	36 (23.8)		
Runny nose (%)			0.007	0.366
No	96 (85.7)	107 (70.9)		
Yes	16 (14.3)	44 (29.1)		
Diarrhea (%)			0.011	0.372
No	110 (98.2)	135 (89.4)		
Yes	2 (1.8)	16 (10.6)		
Nausea (%)			0.084	0.256
No	108 (96.4)	136 (90.1)		
Yes	4 (3.6)	15 (9.9)		
Vomit (%)			1	0.026
No	106 (94.6)	142 (94.0)		
Yes	6 (5.4)	9 (6.0)		
Headaches (%)			<0.001	0.518
No	100 (89.3)	104 (68.9)		
Yes	12 (10.7)	47 (31.1)		
Pneumonia (%)			1	0.003
No	106 (94.6)	143 (94.7)		
Yes	6 (5.4)	8 (5.3)		

### Factors associated with omicron infection and long viral shedding duration among pairs

**Figure 1:**
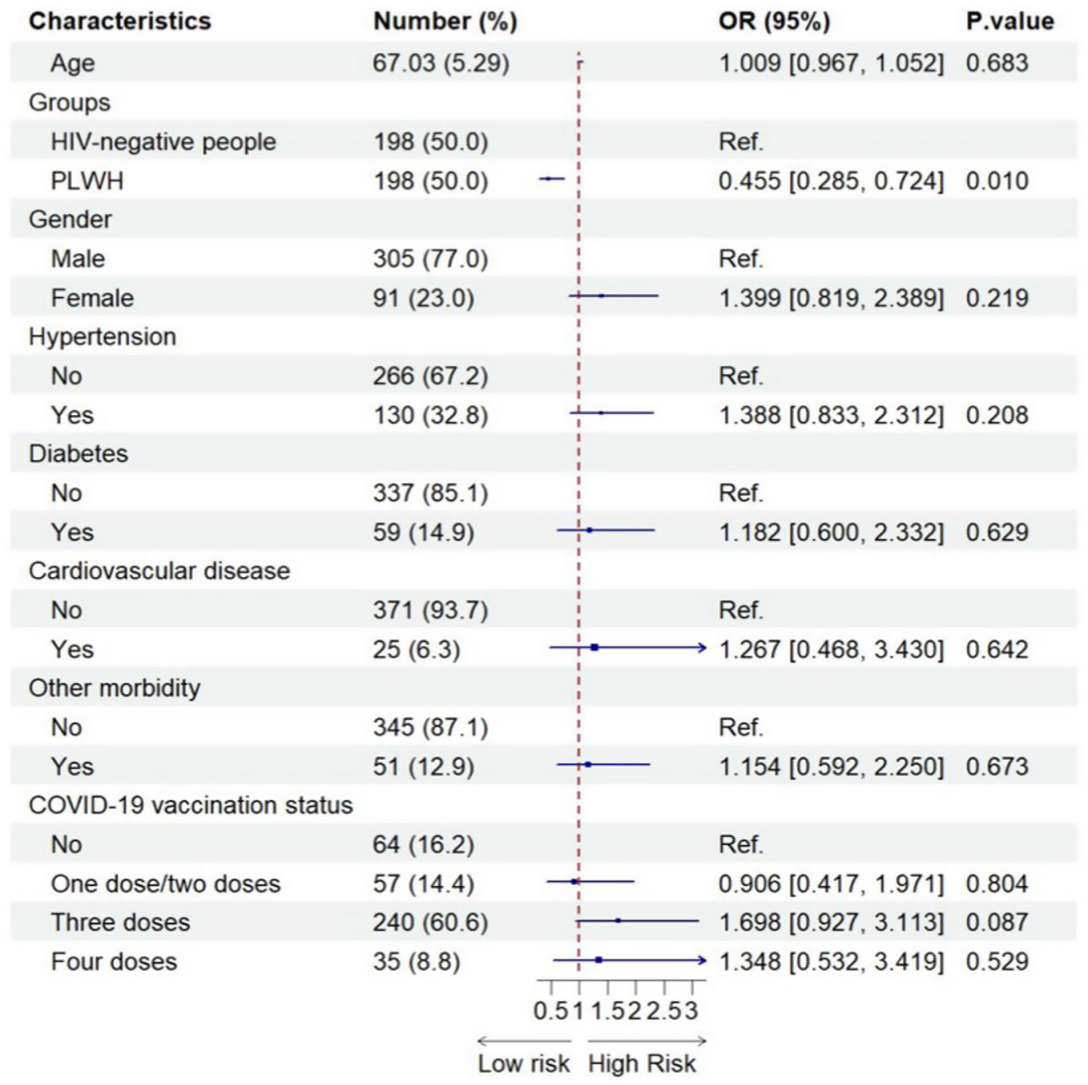
Factors associated with SARS-CoV-2 infection among the combined PLWH and HIV-negative sample (*N* = 396). 396 included all older people after using propensity-score-matching.

Figure 1 shows the factors associated with Omicron infection among the different groups after adjusting for sex and age. Only PLWH were found to be significantly associated with a lower risk of Omicron infection (Figure 1).

**Figure 2:**
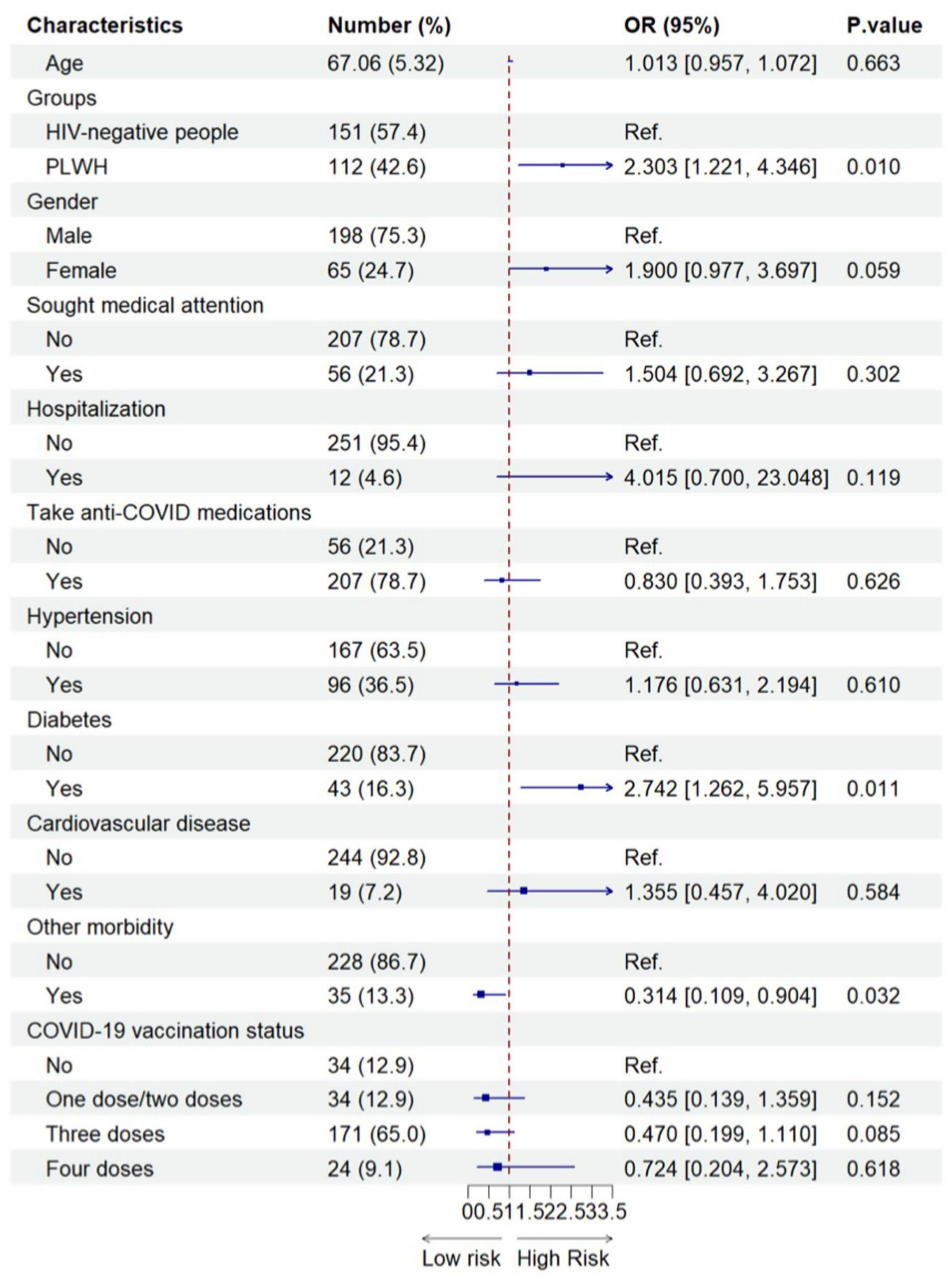
Factors associated with the long viral shedding duration among older SARS-CoV-2 infected participants (*N*=263). 263 included all older people with SARS-CoV-2 infection after propensity-score-matching.

As shown in Figure 2, compared with older HIV-negative people, older PLWH experienced a significantly higher risk of long viral shedding duration after their SARS-CoV-2 infection (adjusted OR = 2.303, 95% CI = 1.221–4.346). In addition, older people who had diabetes had an increased likelihood of long viral shedding duration after the Omicron infection (adjusted OR = 2.742, 95% CI = 1.262–5.957) compared with older people without diabetes. However, interestingly, older people with another comorbidity (i.e., the presence of chronic obstructive pulmonary disease, chronic kidney disease, oncology/cancer, and others) may be a protective factor for the long viral shedding duration after the Omicron infection (adjusted OR = 0.314, 95% CI = 0.109–0.904) (Figure 2).

The findings from the sensitivity analysis also revealed that HIV infection continued to pose as a risk factor for prolonged viral shedding, whereas diabetes exhibited only a marginal influence on delayed viral clearance (Supplementary Table 5).

### Factors associated with omicron infection and long viral shedding duration among all old PLWH and HIV-negative people, respectively

**Figure 3:**
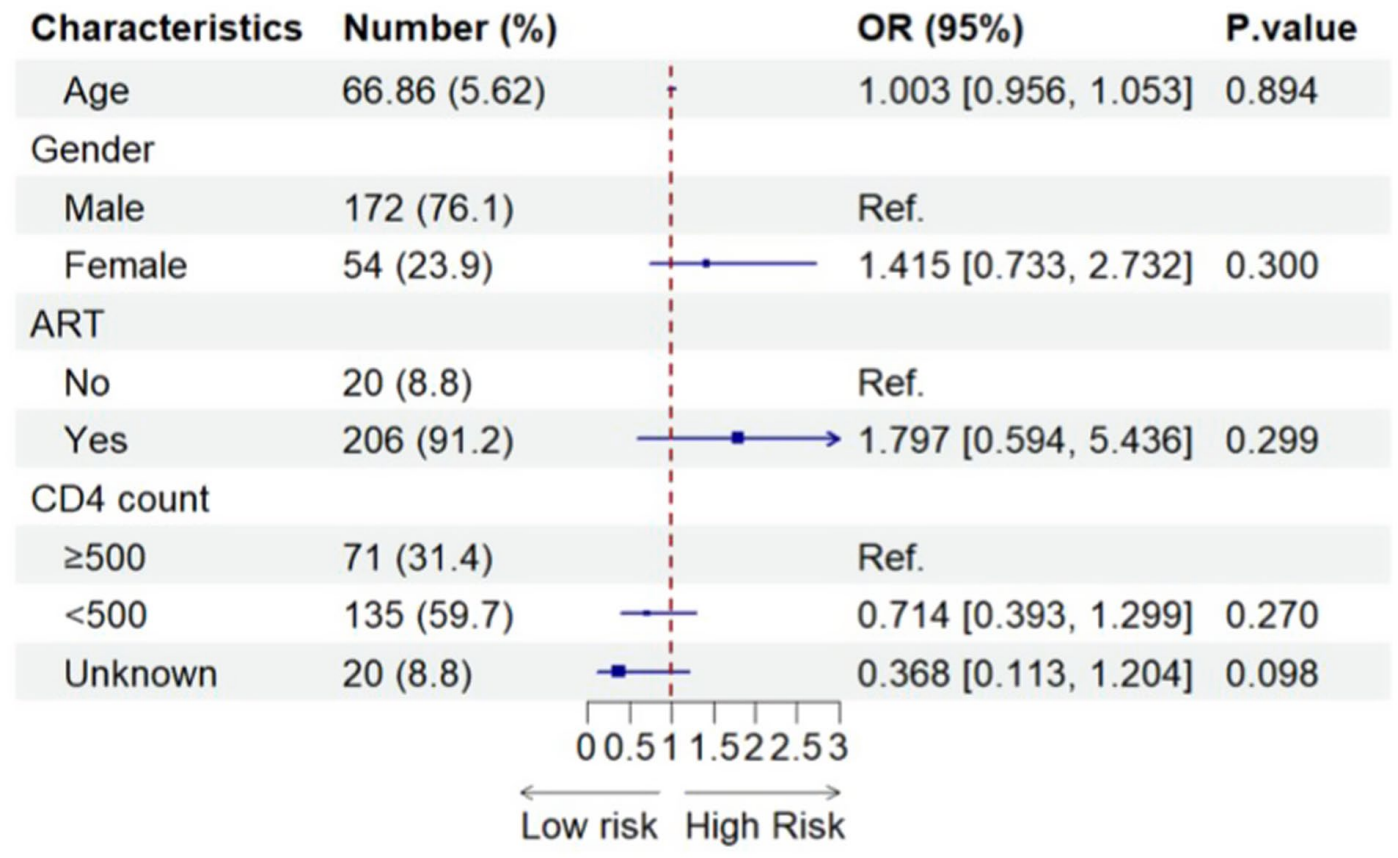
Factors associated with SARS-CoV-2 infection among all older PLWH (*N* = 226). 226 included all older people with SARS-CoV-2 infection before using propensity-score-matching.

The sociodemographic characteristics of older PLWH are shown in Supplementary Table 1, including all participants before PSM. Significant differences were observed between PLWH who had never been infected with the Omicron variant and PLWH who had been infected with the Omicron variant in terms of ART and CD4 counts (Supplementary Table 1). As shown in Figure 3, there was no significant association between ART or CD4 and the odds of having Omicron infection among all older PLWH.

Supplementary Table 2 illustrates the characteristics (demographics, medical utilization, medical conditions, and vaccination status) of all older PLWH infected with the Omicron variant. There were no significant differences between older PLWH with or without long viral shedding duration on any of the variables assessed (Supplementary Table 2). Similarly, no significant differences were found between all old HIV-negative individuals with or without infection with the Omicron variant on these same characteristics (Supplementary Table 3). Supplementary Table 4 presents the assessed characteristics of all old HIV-negative people infected with the Omicron variant. We found significant differences between those with less than and greater than 2 weeks of viral shedding duration in age, seeking medical attention, hospitalization, diabetes, and COVID-19 vaccination status between old HIV-negative people with or without a long viral shedding duration (Supplementary Table 4).

**Figure 4:**
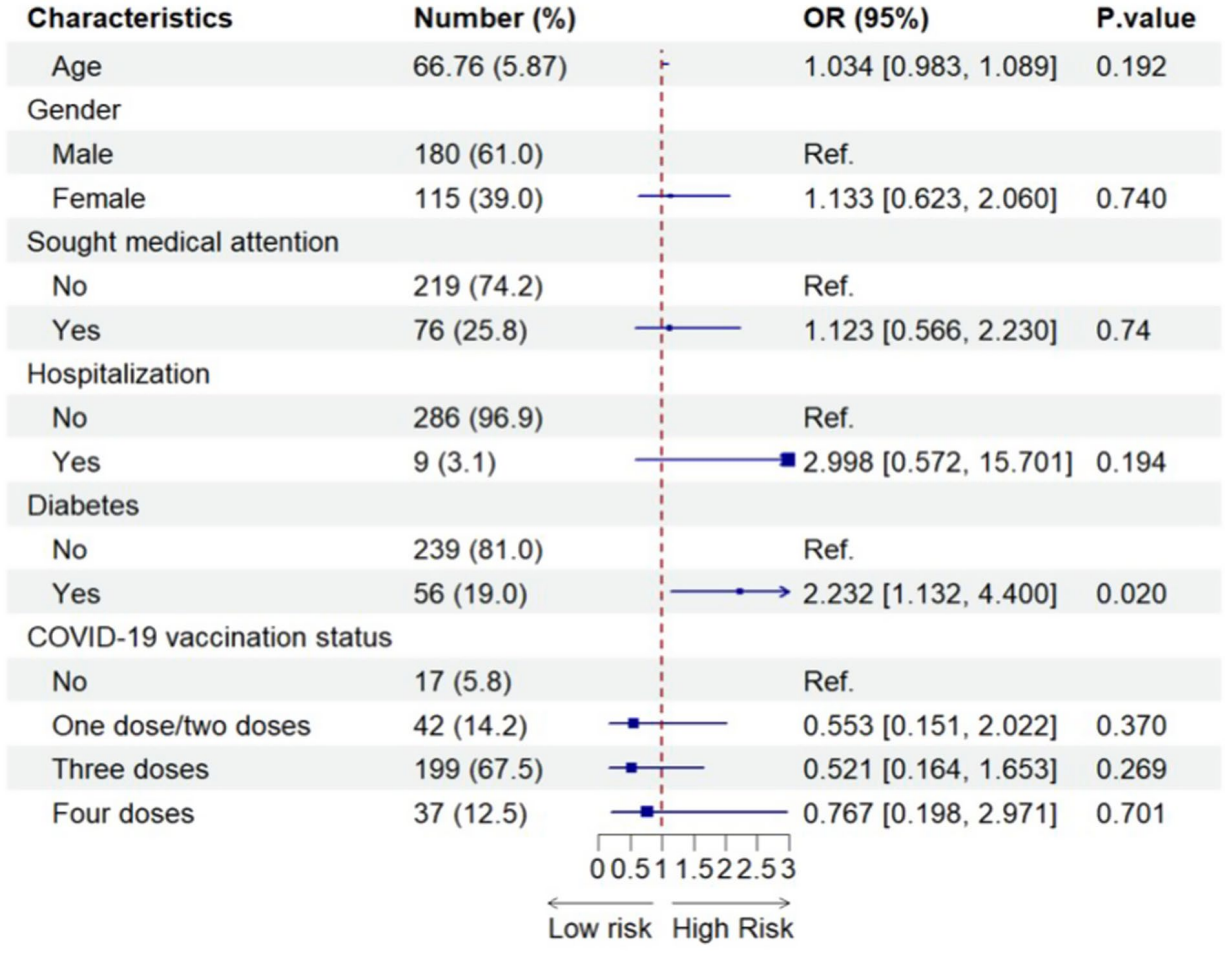
Factors associated with the long viral shedding duration after SARS-CoV-2 infection among all older HIV-negative participants (*N*=295). 295 included all older HIV-negative people with SARS-CoV-2 infection before using propensity-score-matching.

Figure 4 shows the results of the associations between a range of potential risk/protective factors and the long viral shedding duration after SARS-CoV-2 infection among all older HIV-negative individuals before using propensity score matching, after adjusting for potential confounders. The only significant finding was that older HIV-negative individuals who had diabetes experienced greater than twice the odds of the long viral shedding duration compared with older HIV-negative people who did not have diabetes (adjusted OR 2.232, 95% CI = 1.132–4.400).

## Discussion

To our knowledge, this is the first cross-sectional study of older PLWH (i.e., aged 60 years or older) and older HIV-negative people in China to estimate the impact of HIV infection on Omicron infection and viral shedding duration in older people following the Chinese government’s announcement of the ending of the “zero COVID” strategy. We found that older PLWH had a significantly lower prevalence of Omicron infection than older HIV-negative individuals. In addition, in older people, PLWH status was associated with an increased risk of long viral shedding duration after Omicron infection compared to HIV-negative status. Furthermore, diabetes was identified as a risk factor for a long viral shedding duration in this older population with or without HIV infection. However, we did not observe a significant effect of COVID-19 vaccination, ART, CD4+, or HIV viral load on the risk of Omicron infection.

The finding that the prevalence of Omicron infection was lower among older PLWH than among older HIV-negative people is consistent with other findings across all age groups. A cross-sectional study conducted by Tan et al. in Wuhan, China, after the end of the “zero COVID” strategy showed that the prevalence of COVID-19 in PLWH and the general population was 77.5 and 85.3%, respectively, and their mean age was 36 years old (35). Similarly, a study performed by Wu et al., also in Wuhan, China, in May 2020, revealed that the rate of SARS-CoV-2 infection was significantly lower among PLWH than among HIV-negative participants, and their age ranges were 29–50 and 37–58, respectively (36). Furthermore, some prospective cohort studies from developed countries have also found that HIV infection is associated with a lower rate of SARS-CoV-2 infection compared with the general population (37, 38). As such, our finding of a higher prevalence of COVID-19 in older HIV-negative people than in older PLWH is in agreement with previous studies. However, when interpreting this finding, there are a number of considerations to be mindful of: (1) older PLWH demonstrate a lower rate of undergoing COVID-19 testing (39), (2) the number of COVID-19 cases among PLWH may be underestimated primarily due to their reluctance to access COVID-19 testing services (40, 41), (3) asymptomatic SARS-CoV-2 infection may be more common in PLWH than HIV-negative people (42, 43), and (4) PLWH maintain physical distancing due to self-perception of higher SARS-CoV-2 risk (34).

It is worth noting that the odds of being infected with the Omicron variant were lower in our sample of older adults compared with the results from Wuhan, China, likely because of the younger mean age of the participants in the study (36). After China implemented the “Ten New Measures” to optimize its prevention and control measures for COVID-19, most younger people returned to their occupational and recreational activities, while retired older adults may have been more likely to remain in their homes, thus avoiding exposure to the virus.

The second major finding from our study was that older PLWH had an increased risk of long viral shedding duration after Omicron infection compared to older HIV-negative people. This is consistent with the findings from a prospective cohort study at 20 hospitals in South Africa that revealed that the viral shedding time of SARS-CoV-2 in PLWH was longer than that in HIV-negative individuals (median 27 days [interquartile range (IQR) 8–43] vs. 7 days [IQR 4–13]) (44). Similarly, a multicenter prospective study performed in the United States also found that people who were immunocompromised, like those infected with HIV, had longer SARS-CoV-2 shedding durations compared with the general population (45). Furthermore, a review study conducted by Höft also concluded that the median duration of viral shedding was longer in HIV-uninfected individuals (46). These results parallel our findings regarding PLWH experiencing a longer viral shedding duration after Omicron infection than the HIV-negative population. One leading reason for the longer viral shedding duration in PLWH may be that HIV infection is characterized by low CD4 + T-cell counts, and the ensuing immune deficits in adaptive immunity may lead to an inability to eliminate Omicron (47, 48). Furthermore, there were sex-based differences in Omicron viral shedding between PLWH and the HIV-negative older population, which likely stemmed from a multifaceted interplay of biological, immunological, and behavioral factors. Biologically, estrogen in females may enhance antiviral immune responses and reduce viral replication, whereas testosterone in males could suppress immune efficiency, prolonging shedding (49). Immunologically, females often exhibit stronger innate and adaptive immunity, whereas males may experience hyperinflammation and delayed viral clearance (50, 51). Behaviorally, females tend to adhere more strictly to preventive measures and seek healthcare promptly, reducing transmission risks, whereas males may delay isolation or testing (52). Comorbidities such as diabetes and obesity, which are more prevalent in males (53–56), further exacerbate prolonged shedding. In PLWH, these sex-based disparities may be amplified by HIV-related immune dysfunction and interactions with antiretroviral therapy, although similar hormonal and metabolic influences apply to HIV-negative individuals (57, 58). More studies are required to understand these dynamics to inform gender-specific strategies to mitigate transmission in both populations.

Our study also indicated that diabetes is a risk factor for prolonged viral shedding after Omicron infection. In a Swiss cohort, the presence of diabetes in patients increased the risk of prolonged SARS-CoV-2 RNA (59). A retrospective cohort study involving 162 patients conducted by Arfijanto in Indonesia also revealed that people with type 2 diabetes mellitus had a significantly higher risk of experiencing a longer duration of viral shedding after SARS-CoV-2 infection (60). All of the aforementioned evidence collectively supports the existence of an association between diabetes and an extended viral shedding duration following infection with the Omicron variant. In human monocytes, elevated blood sugar levels directly enhance the replication of SARS-CoV-2 (61). Glycolysis sustains the replication of SARS-CoV-2 via the generation of reactive oxygen species (ROS) and activation of hypoxia-inducible factor 1α. Additionally, hyperglycemia can elevate plasma osmotic pressure and impair chemotactic activity, phagocytosis, and intracellular killing capabilities of leukocytes, thereby compromising the body’s resistance to infection and weakening its response to viral invasion (62, 63). Therefore, hyperglycemia serves as a favorable environment for viral proliferation. As a result, older adults individuals with diabetes who contract COVID-19 may require prolonged infection prevention measures during this unique period.

It is noteworthy that our study did not reveal any significant associations between HIV-related characteristics, such as CD4 count, HIV-VL, and ART, and the occurrence of Omicron infection or prolonged viral shedding duration in older people. This finding aligns with the outcomes of a multicenter case-series study conducted in Madrid among HIV patients, which also indicated that SARS-CoV-2 infection in this population was unrelated to HIV-associated clinical parameters (64). Similarly, a population-based cohort study performed by Huang et al. in Wuhan, China, reported the absence of an association between HIV-related characteristics and SARS-CoV-2 infection in PLWH (65). However, this study did not include older PLWH. Our findings, therefore, extend the results of previous studies, supporting that HIV-related variables, namely CD4 count, HIV-VL, and ART, may not exert an influence on Omicron infection or extended viral shedding duration in older PLWH.

Intriguingly, our study revealed that the administration of COVID-19 vaccines did not mitigate the likelihood of contracting SARS-CoV-2 in either group. This finding contradicts the accumulating evidence that highlights COVID-19 vaccination as a pivotal factor in mitigating the incidence of SARS-CoV-2 infection among older adults (66–69). However, the effectiveness of COVID-19 vaccination against infection might decrease more rapidly in individuals with underlying health conditions, particularly among older adults and those with compromised immune systems, such as HIV infection, and decrease significantly 6 months after primary vaccination (66, 70–74). In addition, after the Chinese government ended the “zero COVID” strategy, the Omicron variant prevailed and exhibited a robust immune escape capability, leading to a reduction in vaccine efficacy (75–77). The confluence of these factors may have resulted in the waning of vaccine effectiveness among vulnerable groups.

Our study has several strengths worth noting. First, this study collected data immediately after the Chinese government implemented the “Ten New Measures,” which may decrease the recall bias. In addition, we used propensity matching to balance the sociodemographic characteristics of the participants prior to the analysis. This allowed a stronger comparison between PLWH and HIV-negative samples by reducing the differences in potential covariates. This study has several limitations that need to be considered when interpreting the findings. First, the cross-sectional study method limited the causal inferences. Second, information on the characteristics of SARS-CoV-2 infection and the viral shedding duration was collected using a self-report questionnaire. Therefore, the incidence of SARS-CoV-2 infection and duration of viral shedding may lead to either an overestimation or underestimation of the reported outcomes. Future studies should incorporate objective verification methods (e.g., PCR testing and electronic health records) to minimize recall bias and strengthen the validity of the findings. Third, because all participants were recruited exclusively from Ningbo, the generalizability of our findings to older adults in other regions of China may be limited. The results should be interpreted with caution, as they may reflect specific local demographic, socioeconomic, or cultural conditions unique to Ningbo during the post-“zero-COVID” transition period. Further studies involving diverse geographic populations are required to validate these findings in a broader context. Fourth, it is possible that residual confounders, such as lifestyle behaviors not measured, including smoking, drinking, exercising, and medication history, may have influenced the findings. Fifth, because of the relatively small sample size, statistically significant results should be interpreted with caution. Finally, detailed vaccination data—including the time elapsed since the last dose, vaccine type (e.g., mRNA, viral vector, or inactivated vaccines), and booster sequence—were not collected in our study due to limitations in the questionnaire design. These factors can significantly influence infection risk, particularly in older or immunocompromised populations. Therefore, future studies should incorporate these variables to enhance the robustness of our findings.

## Conclusion

In summary, our study delineates the clinical symptoms of Omicron infections in both old PLWH and old HIV-negative individuals, identifies different factors associated with SARS-CoV-2 infection and viral shedding duration, and identifies the adverse effects of diabetes. These insights can guide clinical and public health decision-making regarding COVID-19 during this special period in China. To effectively anticipate and counter potential future outbreaks of disease X, it is imperative to establish extensive prospective cohorts that can delineate vulnerabilities among different populations, particularly older individuals with comorbid conditions or compromised immune systems. Additionally, an examination of viral kinetics is crucial in order to gain a deeper understanding and insights into the disease’s progression and characteristics.

## Data Availability

The raw data supporting the conclusions of this article will be made available by the authors, without undue reservation.
