# Supplementary material for: Are older adults living with HIV more susceptible to omicron infection compared to their HIV-negative peers in China: a cross-sectional study
Source: Front Public Health. 2025 Nov 5;13:1685868. doi: 10.3389/fpubh.2025.1685868 (PMC12627037; doi:10.3389/fpubh.2025.1685868)
Supplement: Supplementary file 1 [file Supplementary_file_1.docx]

**Survey of the COVID-19 infection situation in the general population and special populations**

***Hello and welcome to the survey, which aims to understand the COVID-19 infection so that we can provide more targeted health education, vaccination, and treatment services in the future. Thank you for your participation and cooperation.***

**Investigative Agency:**

**Control ID:**

**1. Age:**

**2. Gender:**

(1) Male

(2) Female

**3. Marital status:**

(1) Single

(2) Married

**4. Education level:**

(1) Junior high school or lower

(2) High school

(3) College or higher

**5. Since December 7, 2022, have you experienced any of the following symptoms (multiple choices are allowed):**

(1) Fever (body temperature ≥ 37.3°C)

(2) Fatigue

(3) Muscle aches

(4) Cough

(5) Runny nose

(6) Diarrhea

(7) Nausea

(8) Vomiting

(9) Headache

(10) Pneumonia

(11) No symptoms above

**6. Have you been infected with the COVID-19 since December 7, 2022?**

(1) Yes

(2) No

**7. Have you ever sought medical treatment after being infected with the COVID-19? (If you select no, skip to 9)?**

(1) Yes

(2) No

**8. Have you been hospitalized since you were infected with the COVID-19?**

(1) Yes

(2) No

**9. After you are infected with the COVID-19, the maximum body temperature is:**

(1) >39°C

(2) 38-39°C

(3) 37.3-38°C

(4) <37.3°C

**10. Do you take anti-COVID medications after COVID-19 infection?**

(1) Yes

(2) No

**11. Weeks about your COVID-19 antigen or nucleic acid to turn negative?**

(1) < 1 week

(2) 1-2 weeks

(3) 2-3 weeks

(4) > 3 weeks

**12. Do you have any of the following underlying medical conditions?**

(1) Hypertension

(2) Diabetes mellitus

(3) Chronic obstructive pulmonary disease

(4) Cardiovascular disease

(5) Chronic kidney disease

(6) Oncology/cancer

(7) Cerebrovascular diseases

(8) Others

**13. Have you been vaccinated against the COVID-19?**

(1) Yes, you have received _ doses.

(2) No.

eTable 1 Characteristics of all old PLWH

| **Characteristics** | **COVID-19 Infection** | | *P* | SMD |
| --- | --- | --- | --- | --- |
|  | Never Infected (*N* = 97) | Infected  (N = 129) |  |  |
| *Age (mean (SD))* | 66.76 (5.12) | 66.93 (5.99) | 0.825 | 0.030 |
| *Gender (%)* |  |  | 0.399 | 0.136 |
| Male | 77 (79.4) | 95 (73.6) |  |  |
| Female | 20 (20.6) | 34 (26.4) |  |  |
| *ART (%)* |  |  | 0.064 | 0.276 |
| No | 13 (13.4) | 7 (5.4) |  |  |
| Yes | 84 (86.6) | 122 (94.6) |  |  |
| *CD4 count (%)* |  |  | 0.057 | 0.320 |
| ≥500 | 25 (25.8) | 46 (35.7) |  |  |
| <500 | 59 (60.8) | 76 (58.9) |  |  |
| Unknown | 13 (13.4) | 7 (5.4) |  |  |
| *HIV viral load (%)* |  |  | 0.182 | 0.245 |
| Undetectable | 73 (75.3) | 105 (81.4) |  |  |
| Detectable | 8 (8.2) | 13 (10.1) |  |  |
| Unknown | 16 (16.5) | 11 (8.5) |  |  |
| *Marital status (%)* |  |  | 0.803 | 0.109 |
| Single | 2 (2.1) | 1 (0.8) |  |  |
| Married | 95 (97.9) | 128 (99.2) |  |  |
| *Registered residence (%)* |  |  | 0.781 | 0.066 |
| Ningbo | 87 (89.7) | 113 (87.6) |  |  |
| Others | 10 (10.3) | 16 (12.4) |  |  |
| *Race (%)* |  |  | 1.000 | 0.027 |
| Han | 96 (99.0) | 128 (99.2) |  |  |
| Minority | 1 (1.0) | 1 (0.8) |  |  |
| *Education level (%)* |  |  | 0.815 | 0.086 |
| Junior high school or lower | 79 (81.4) | 108 (83.7) |  |  |
| High school | 14 (14.4) | 15 (11.6) |  |  |
| College or higher | 4 (4.1) | 6 (4.7) |  |  |
| *Hypertension (%)* |  |  | 0.158 | 0.214 |
| No | 78 (80.4) | 92 (71.3) |  |  |
| Yes | 19 (19.6) | 37 (28.7) |  |  |
| *Diabetes (%)* |  |  | 0.304 | 0.168 |
| No | 88 (90.7) | 110 (85.3) |  |  |
| Yes | 9 (9.3) | 19 (14.7) |  |  |
| *Cardiovascular disease (%)* |  |  | 0.446 | 0.148 |
| No | 94 (96.9) | 121 (93.8) |  |  |
| Yes | 3 (3.1) | 8 (6.2) |  |  |
| *Other morbidity (%)* |  |  | 0.389 | 0.144 |
| No | 86 (88.7) | 108 (83.7) |  |  |
| Yes | 11 (11.3) | 21 (16.3) |  |  |
| *COVID-19 vaccination status (%)* |  |  | 0.155 | 0.310 |
| No | 29 (29.9) | 27 (20.9) |  |  |
| One dose/two doses | 16 (16.5) | 14 (10.9) |  |  |
| Three doses | 48 (49.5) | 83 (64.3) |  |  |
| Four doses | 4 (4.1) | 5 (3.9) |  |  |

eTable 2 Characteristics of all old PLWH who infected COVID-19

| **Characteristics** | **the viral shedding duration** | | *P* | SMD |
| --- | --- | --- | --- | --- |
|  | < 2 weeks  (*N* = 76) | ≥ 2 weeks  (*N* = 53) |  |  |
| *Age (mean (SD))* | 66.64 (5.52) | 67.34 (6.64) | 0.519 | 0.114 |
| *Gender (%)* |  |  | 0.304 | 0.219 |
| Male | 59 (77.6) | 36 (67.9) |  |  |
| Female | 17 (22.4) | 17 (32.1) |  |  |
| *ART (%)* |  |  | 1.000 | 0.017 |
| No | 4 (5.3) | 3 (5.7) |  |  |
| Yes | 72 (94.7) | 50 (94.3) |  |  |
| *CD4 count (%)* |  |  | 0.994 | 0.020 |
| ≥500 | 27 (35.5) | 19 (35.8) |  |  |
| <500 | 45 (59.2) | 31 (58.5) |  |  |
| Unknown | 4 (5.3) | 3 (5.7) |  |  |
| *HIV viral load (%)* |  |  | 0.709 | 0.151 |
| Undetectable | 61 (80.3) | 44 (83.0) |  |  |
| Detectable | 9 (11.8) | 4 (7.5) |  |  |
| Unknown | 6 (7.9) | 5 (9.4) |  |  |
| *Marital status (%)* |  |  | 1.000 | 0.163 |
| Single | 1 (1.3) | 0 (0.0) |  |  |
| Married | 75 (98.7) | 53 (100.0) |  |  |
| *Registered residence (%)* |  |  | 0.968 | 0.056 |
| Ningbo | 66 (86.8) | 47 (88.7) |  |  |
| Others | 10 (13.2) | 6 (11.3) |  |  |
| *Race (%)* |  |  | 1.000 | 0.163 |
| Han | 75 (98.7) | 53 (100.0) |  |  |
| Minority | 1 (1.3) | 0 (0.0) |  |  |
| *Education level (%)* |  |  | 0.732 | 0.143 |
| Junior high school or lower | 62 (81.6) | 46 (86.8) |  |  |
| High school | 10 (13.2) | 5 (9.4) |  |  |
| College or higher | 4 (5.3) | 2 (3.8) |  |  |
| *Sought medical attention after COVID-19 infection (%)* |  |  | 0.154 | 0.294 |
| No | 66 (86.8) | 40 (75.5) |  |  |
| Yes | 10 (13.2) | 13 (24.5) |  |  |
| *Hospitalization after COVID-19 infection (%)* |  |  | 0.113 | 0.330 |
| No | 72 (94.7) | 45 (84.9) |  |  |
| Yes | 4 (5.3) | 8 (15.1) |  |  |
| *Took anti-COVID medications after COVID-19 infection (%)* |  |  | 0.853 | 0.068 |
| No | 22 (28.9) | 17 (32.1) |  |  |
| Yes | 54 (71.1) | 36 (67.9) |  |  |
| *Hypertension (%)* |  |  | 1.000 | 0.014 |
| No | 54 (71.1) | 38 (71.7) |  |  |
| Yes | 22 (28.9) | 15 (28.3) |  |  |
| *Diabetes (%)* |  |  | 0.392 | 0.196 |
| No | 67 (88.2) | 43 (81.1) |  |  |
| Yes | 9 (11.8) | 10 (18.9) |  |  |
| *Cardiovascular disease (%)* |  |  | 0.368 | 0.221 |
| No | 73 (96.1) | 48 (90.6) |  |  |
| Yes | 3 (3.9) | 5 (9.4) |  |  |
| *Other morbidity (%)* |  |  | 0.302 | 0.234 |
| No | 61 (80.3) | 47 (88.7) |  |  |
| Yes | 15 (19.7) | 6 (11.3) |  |  |
| *COVID-19 vaccination status (%)* |  |  | 0.641 | 0.231 |
| No | 13 (17.1) | 14 (26.4) |  |  |
| One dose/two doses | 9 (11.8) | 5 (9.4) |  |  |
| Three doses | 51 (67.1) | 32 (60.4) |  |  |
| Four doses | 3 (3.9) | 2 (3.8) |  |  |

eTable 3 Characteristics of all old HIV-negative people

| **Characteristics** | COVID-19 Infection | | *P* | SMD |
| --- | --- | --- | --- | --- |
|  | Never Infected (*N* = 85) | Infected  (*N* = 295) |  |  |
| Age (mean (SD)) | 67.40 (6.94) | 66.76 (5.87) | 0.394 | 0.100 |
| *Gender (%)* |  |  | 0.923 | 0.027 |
| Male | 53 (62.4) | 180 (61.0) |  |  |
| Female | 32 (37.6) | 115 (39.0) |  |  |
| *Marital status (%)* |  |  | 1.000 | 0.015 |
| Single | 1 (1.2) | 3 (1.0) |  |  |
| Married | 84 (98.8) | 292 (99.0) |  |  |
| *Registered residence (%)* |  |  | 0.760 | 0.068 |
| Ningbo | 79 (92.9) | 279 (94.6) |  |  |
| Others | 6 (7.1) | 16 (5.4) |  |  |
| *Race (%)* |  |  | NA | <0.001 |
| Han | 85 (100.0) | 295 (100.0) |  |  |
| Minority | 0 (0.0) | 0 (0.0) |  |  |
| *Education level (%)* |  |  | 0.572 | 0.134 |
| Junior high school or lower | 68 (80.0) | 220 (74.6) |  |  |
| High school | 11 (12.9) | 51 (17.3) |  |  |
| College or higher | 6 (7.1) | 24 (8.1) |  |  |
| *Hypertension (%)* |  |  | 0.429 | 0.113 |
| No | 52 (61.2) | 164 (55.6) |  |  |
| Yes | 33 (38.8) | 131 (44.4) |  |  |
| *Diabetes (%)* |  |  | 1.000 | 0.004 |
| No | 69 (81.2) | 239 (81.0) |  |  |
| Yes | 16 (18.8) | 56 (19.0) |  |  |
| *Cardiovascular disease (%)* |  |  | 0.739 | 0.076 |
| No | 81 (95.3) | 276 (93.6) |  |  |
| Yes | 4 (4.7) | 19 (6.4) |  |  |
| *Other morbidity (%)* |  |  | 0.477 | 0.109 |
| No | 75 (88.2) | 270 (91.5) |  |  |
| Yes | 10 (11.8) | 25 (8.5) |  |  |
| *COVID-19 vaccination status (%)* |  |  | 0.185 | 0.264 |
| No | 4 (4.7) | 17 (5.8) |  |  |
| One dose/two doses | 19 (22.4) | 42 (14.2) |  |  |
| Three doses | 48 (56.5) | 199 (67.5) |  |  |
| Four doses | 14 (16.5) | 37 (12.5) |  |  |

eTable 4 Characteristics of all old HIV-negative people who infected COVID-19

| **Characteristics** | **the viral shedding duration** | | *P* | SMD |
| --- | --- | --- | --- | --- |
|  | < 2 weeks  (*N* = 228) | ≥ 2 weeks  (*N* = 67) |  |  |
| *Age (mean (SD))* | 66.25 (5.25) | 68.48 (7.42) | 0.006 | 0.347 |
| *Gender (%)* |  |  | 0.860 | 0.044 |
| Male | 138 (60.5) | 42 (62.7) |  |  |
| Female | 90 (39.5) | 25 (37.3) |  |  |
| *Marital status (%)* |  |  | 1.000 | 0.057 |
| Single | 2 (0.9) | 1 (1.5) |  |  |
| Married | 226 (99.1) | 66 (98.5) |  |  |
| *Registered residence (%)* |  |  | 1.000 | 0.031 |
| Ningbo | 216 (94.7) | 63 (94.0) |  |  |
| Others | 12 (5.3) | 4 (6.0) |  |  |
| *Race (%)* |  |  | NA | <0.001 |
| Han | 228 (100.0) | 67 (100.0) |  |  |
| Minority | 0 (0.0) | 0 (0.0) |  |  |
| *Education level (%)* |  |  | 0.345 | 0.216 |
| Junior high school or lower | 166 (72.8) | 54 (80.6) |  |  |
| High school | 41 (18.0) | 10 (14.9) |  |  |
| College or higher | 21 (9.2) | 3 (4.5) |  |  |
| *Sought medical attention after COVID-19 infection (%)* |  |  | 0.096 | 0.247 |
| No | 175 (76.8) | 44 (65.7) |  |  |
| Yes | 53 (23.2) | 23 (34.3) |  |  |
| *Hospitalization after COVID-19 infection (%)* |  |  | 0.005 | 0.351 |
| No | 225 (98.7) | 61 (91.0) |  |  |
| Yes | 3 (1.3) | 6 (9.0) |  |  |
| *Took* *anti-COVID medications after COVID-19 infection (%)* |  |  | 0.957 | 0.037 |
| No | 30 (13.2) | 8 (11.9) |  |  |
| Yes | 198 (86.8) | 59 (88.1) |  |  |
| *Hypertension (%)* |  |  | 0.295 | 0.165 |
| No | 131 (57.5) | 33 (49.3) |  |  |
| Yes | 97 (42.5) | 34 (50.7) |  |  |
| *Diabetes (%)* |  |  | 0.002 | 0.430 |
| No | 194 (85.1) | 45 (67.2) |  |  |
| Yes | 34 (14.9) | 22 (32.8) |  |  |
| *Cardiovascular disease (%)* |  |  | 1.000 | 0.025 |
| No | 213 (93.4) | 63 (94.0) |  |  |
| Yes | 15 (6.6) | 4 (6.0) |  |  |
| *Other morbidity (%)* |  |  | 0.929 | 0.048 |
| No | 208 (91.2) | 62 (92.5) |  |  |
| Yes | 20 (8.8) | 5 (7.5) |  |  |
| *COVID-19 vaccination status (%)* |  |  | 0.066 | 0.335 |
| No | 9 (3.9) | 8 (11.9) |  |  |
| One dose/two doses | 31 (13.6) | 11 (16.4) |  |  |
| Three doses | 160 (70.2) | 39 (58.2) |  |  |
| Four doses | 28 (12.3) | 9 (13.4) |  |  |

**eTable 5 Factors associated with the long viral shedding duration among older SARS-CoV-2 infected participants using multivariate logistic regression**

| **Variable** | **B** | **S.E** | **Wald** | **OR (95% CI)** | ***P*** |
| --- | --- | --- | --- | --- | --- |
| HIV-infected Status |  |  |  |  |  |
| HIV-negative People | - | - | - | Ref. |  |
| PLWH | 1.515 | 4.489 | 20.155 | 4.15 (2.22-7.76) | <0.001 |
| Age | 0.03 | 1.119 | 1.252 | 1.03 (0.98-1.09) | 0.211 |
| Gender |  |  |  |  |  |
| Male | - | - | - | Ref. |  |
| Female | 0.24 | 0.669 | 0.447 | 1.13 (0.61-2.12) | 0.697 |
| Sought medical attention |  |  |  |  |  |
| No | - | - | - | Ref. |  |
| Yes | -0.081 | -0.175 | 0.031 | 1.20 (0.62-2.31) | 0.585 |
| Hospitalization |  |  |  |  |  |
| No | - | - | - | Ref. |  |
| Yes | 1.703 | 1.901 | 3.613 | 1.91 (0.56-6.49) | 0.297 |
| Take anti-CPVID medications |  |  |  |  |  |
| No | - | - | - | Ref. |  |
| Yes | -1.379 | -1.387 | 1.925 | 1.06 (0.28-4.03) | 0.934 |
| Hypertension |  |  |  |  |  |
| No | - | - | - | Ref. |  |
| Yes | 0.527 | 0.836 | 0.699 | 1.16 (0.66-2.05) | 0.601 |
| Diabetes |  |  |  |  |  |
| No | - | - | - | Ref. |  |
| Yes | 0.695 | 1.511 | 2.284 | 1.91 (0.96-3.78) | 0.063 |
| Cardiovascular disease |  |  |  |  |  |
| No | - | - | - | Ref. |  |
| Yes | -0.798 | -1.2 | 1.44 | 0.71 (0.30-1.70) | 0.438 |
| Other morbidity |  |  |  |  |  |
| No | - | - | - | Ref. |  |
| Yes | -0.31 | -0.442 | 0.196 | 1.06 (0.61-1.84) | 0.848 |
| COVID-19 vaccination status |  |  |  |  |  |
| No | - | - | - | Ref. |  |
| One dose/two doses | 0.546 | 0.405 | 0.164 | 1.34 (0.11-16.32) | 0.818 |
| Three doses | 0.858 | 1.386 | 1.92 | 2.21 (0.68-7.18) | 0.189 |
| Four doses | 0.079 | 0.178 | 0.032 | 1.03 (0.45-2.39) | 0.942 |

**eTable 6 Results of model diagnostics**

| **Model 1** |  | **Model 2** |  |
| --- | --- | --- | --- |
| **Variable** | **VIF** | **Variable** | **VIF** |
| HIV-infected Status | 1.123177 | HIV-infected Status | 1.158744 |
| Age | 1.057897 | Age | 1.187545 |
| Gender | 1.046406 | Gender | 1.062500 |
| Hypertension | 3.739786 | Sought medical attention | 1.342558 |
| Diabetes | 1.339327 | Hospitalization | 1.840465 |
| Cardiovascular disease | 2.107116 | Take anti-CPVID medications | 1.352624 |
| Other morbidity | 5.241469 | Hypertension | 3.990303 |
| COVID-19 vaccination status | 1.486626 | Diabetes | 1.434199 |
|  |  | Cardiovascular disease | 1.793314 |
|  |  | Other morbidity | 5.204055 |
|  |  | COVID-19 vaccination status | 1.624864 |
|  |  |  |  |
| **R²** | **Value** | **R²** | **Value** |
| Maximum likelihood pseudo -R² | 0.0678 | Maximum likelihood pseudo-R² | 0.1257135 |
| Cragg and Uhler's pseudo-R² | 0.0940 | Cragg and Uhler's pseudo-R² | 0.1755511 |
|  |  |  |  |
| AIC | 523.4465 | AIC | 337.3236 |
| BIC | 567.7576 | BIC | 387.9584 |
|  |  |  |  |
| **Goodness-of-fit tests** | ***P*** | **Goodness-of-fit tests** | ***P*** |
| Hosmer–Lemeshow | 0.895 | Hosmer–Lemeshow | 0.419 |
